# Philadelphia County Medical Society

**Published:** 1891-10

**Authors:** 


### PHILADELPHIA COUNTY MEDICAL SOCIETY.

*Stated Meeting, June* 24, 1891.

THE PRESIDENT, JOHN B. ROBERTS, M. D., in the chair.

Dr. S. MACCUEN SMITH submitted a paper on

### EMPLOYMENT OF PILOCARPINE MURIATE IN LABYRINTHINE DISEASE WITH REPORT OF CASES.

GENTLEMEN : Our object in calling attention to the following interesting cases is, as far as possible, to determine the diseases in which pilocarpine may be of decided benefit, and, if possible, to add to its sphere of usefulness.

CASE I.—Mr. F. W., of New Jersey, 20 years of age, has been a bright scholar until within the last two years, when, on account of progressive loss of hearing, he was compelled to leave school and seek medical aid. From a critical examination of the patient, and also of his parents, the personal and family history were pronounced unusually good. The father gave positive assurance of being entirely exempt from any previous specific history, and certainly observation would substantiate his statement, as his entire family of seven children showed impressive evidence of good health.

The patient stated that he had been under treatment for three years with several specialists in neighboring cities, only to meet with discouragement, as his hearing continued to grow worse.

On examination the external ear and canal were found to be normal ; membrana tympani slightly opaque and retracted, otherwise normal, except some inflammation had extended to the manubrium mallei. In the post-nasal space the pharyngeal tonsils were found to be much enlarged, the hypertrophy extending beyond and occluding each eustachian tube. Very low notes of both aërial and osseous conduction of sound were perceived in each ear—the higher notes not being heard except when intense—which would seem to prove that the internal ear was at fault ; and as the voice could be heard much better than the watch-tick, this would offer additional evidence of internal ear disease. By my watch, which measures fifty inches, the hearing distance of R. E. $= \frac{1}{L}$ ; of L. E, = quite negative ; and yet ordinary conversation from the base voice could be heard at six feet, but individual words could not be distinguished until the sound-wave was intensified.

By removing the enlarged tonsils and post-nasal adenoid growths, the hearing power was somewhat increased by equalizing the atmospheric pressure, and this to an extent correcting the retracted condition of the membrana tympani. Knowing this patient to have been under special treatment for some time, and that the usual methods of procedure had failed to give relief, we deemed a repetition of the same to be a useless experiment, and therefore concluded to place him under the pilocarpine muriate treatment, by hypodermic injections. As the object of such treatment is to produce profuse diaphoresis, the dose to be employed must be in accordance with individual idiosyncrasy. It has been our custom to commence with a small dose, one-sixteenth to one-eighth grain, and gradually increase, until the full physiological effects of the drug are produced, provided contra-indications (*to be determined by previous careful examination*) do not manifest themselves.

The dose usually employed is one-sixth grain, although one-fourth grain is frequently necessary. In view of the danger to life that is possible to occur in administering full doses of pilocarpine, Dr. Lawrence Turnbull and other authors advise the use of atropine or strychnia in conjunction with pilocarpine. This is certainly a proper and safe precaution, and should be employed in selected cases; yet, in the greater number of cases, the writer does not find this essential.

As patients cannot usually stand the daily injection, it is our custom to administer one hypodermic once every second day; always *insisting* on the patient remaining in *bed* from three to five hours after each treatment, as the perspiration continues for that length of time, and any undue exposure or exertion may produce unfavorable symptoms. These hypodermics are continued until five, eight, or ten have been given, and then, if additional ones are required, they may be administered at intervals of five to ten days, as the symptoms indicate.

We will now relate the treatment and improvement of the case in question, the details of which are purposely given, as they are intended to express in main the general treatment and improvement of the subsequent cases; the points of difference, however, will be mentioned in each case.

February 2, 1869, we gave the above case the first hypodermic of one-eighth grain (the hearing distance, you will remember, was R. E. $\frac{1}{L}$, L. E. negative); next day patient thought tinnitus was much less severe; no improvement in hearing; perspiration not profuse. February 4th, gave hypodermic of one-sixth grain, which caused copious diaphoresis, some nausea, and headache. During that evening patient heard wagons passing his window, but could not hear the music from some stringed instruments only fifteen or twenty feet away; his hearing for conversation had materially increased; tinnitus about the same; watch-tick R. E. $\frac{2}{L}$, L. E. $\frac{4}{L}$. It is interesting to note the marked improvement in left ear, which up to this time had been negative.

*6th.* We repeated hypodermic injection of one-sixth grain, as we found this to produce full physiological effect; perspiration about the same; absence of nausea and headache. The next day patient called and stated his hearing had never been so bad, he being unable to hear anything except very intense *high* notes; he was, therefore, much discouraged; all his prevous hopefulness having vanished, he refused to submit to further treatment. However, he called the following day and informed me he would continue treatment, as "he thought nothing could possibly make him worse." The peculiar effects of this last hypodermic were the almost entire loss of hearing, and the change to perceiving *only high* notes.

*8th.* One-sixth grain was again given, which resulted in the hearing being restored to R. E. $\frac{3}{L}$, L. E. $\frac{3}{L}$; tinnitus almost gone; feeling much better in general health and spirits.

10*th* and 12*th.* He received one-sixth grain, with marked improvement in hearing; R. E. $\frac{9}{L}$, L. E. $\frac{11}{L}$; no tinnitus; general health continues to improve.

At this date, patient's *father* called to be treated for "some fever blisters on tongue and throat," which, on examination, presented such questionable appearances that I, without hesitation, pronounced them syphilitic. He then admitted having had a chancre when twenty years old (his age at this writing being forty-nine). His excuse for previous false statements was that every physician whom his son had heretofore consulted questioned his family history, and a confession on the part of the father invariably resulted in the son being put on large increasing doses of iodide of potassium and mercury, which so impaired his health that the treatment was discontinued; and as his son had never improved under such medicament, he concluded to conceal his family history, with the hope that other methods of treatment might be pursued.

Granting that the patient's impairment of hearing was specific in origin, in view of his marked improvement I thought it wise to continue with the pilocarpine treatment, in order to more thoroughly establish its efficiency in this class of cases.

The hypodermics were continued on February 12th, and every second day thereafter until eight had been given, at which time his hearing distance was, R. E.$\frac{26}{L}$, L. E.$\frac{32}{L}$; general health improving daily; his weight has increased seven pounds; tinnitus aurium entirely gone

21*st*. The patient returned home, feeling that he had entirely recovered; but in two weeks he called again at my office with hearing somewhat impaired—R. E.$\frac{22}{L}$, L. E.$\frac{26}{L}$—which he thought came from "catching cold," due to exposure. I again gave him three hypodermics, his hearing increasing to R. E.$\frac{36}{L}$, L. E.$\frac{39}{L}$. Additional hypodermics did not improve the hearing. Thinking he required some *specific* treatment, and remembering that iodide of potassium and mercury could not be tolerated by his stomach, I prescribed Hostelley's syrup of hydriodic acid in two-drachm doses, four times a day, well diluted in water; also inunctions of one drachm of ung. hydrarg. each night and morning. This treatment was continued for six months without experiencing any inconvenience, his hearing remaining about the same; no tinnitus; general health better than ever before, and at this writing—about fifteen months since beginning treatment—his hearing and general condition continue to be good, although the patient has not been taking any medicine for the past nine months.

Case II. Mr. S. G., of Pennsylvania, aged 71 years, consulted the writer, with his family physician, July 8, 1888, and gave the following history: On January 6th of the same year, when arising at his usual hour, he was much alarmed at not hearing the customary noise on the street; thinking, however, that his servant had by mistake awakened him at too early an hour, he consulted his watch, and, on finding the hour rather later than usual, and as everything appeared distressingly quiet, he realized that his hearing had been entirely lost during the night. This complete loss of hearing was not attended with pain, tinnitis aurium, discharge from the ears, nor inconvenience of any kind.

After being under treatment for three months, and not receiving the slightest benefit, while drinking some *hot* milk by the direction of his physician, he was seized with intense tinnitus aurium, vertigo, and constant emesis *without nausea*, but with entire absence of pain. The emesis continued for only three hours, but the tinnitus and vertigo increased in severity.

In this condition the patient consulted the writer. A more despondent and pitiable mortal could not be imagined, He was not able to walk without assistance, and the tinnitus had prevented sleep for almost two weeks. The membrana tympani and the external auditory canal were normal, excepting the changes peculiar to a person of his years; the post-nasal space, including the Eustachian tubes, were in good condition; in brief, both ears and their appendages presented the usual normal condition in so far as observation alone could determine. In this connection it is interesting to note the entire absence of evidence of disease in such cases. Osseous conduction of sound was well marked, especially in left ear; aërial conduction of sound was quite lost in both ears.

The family physician informed me that his patient had been on large increasing doses of iodide of potassium and mercury, but the stomach would no longer retain the potassium iodide.

We subjected this patient to the pilocarpine treatment, as previously described, with the following results: First treatment resulted in slight decrease in vertigo and tinnitus, but hearing power remains the same; caused some temporary vomiting, which was quite severe for two hours. The second and third hypodermics were given without any appreciable benefit; the fourth and fifth, however, were productive of marked improvement in vertigo and tinnitus, and at the same time caused such changes in the perceptive organs of hearing that he was able to hear intense waves of sound. After the sixth treatment, tinnitus and vertigo had almost ceased, and hearing distance increased to $\frac{1}{L}$ in both ears. The seventh and eighth treatments did not produce much change in hearing distance, but entirely relieved vertigo and tinnitus. The ninth hypodermic caused much improvement in hearing: R. E. $= \frac{9}{L}$, L. E. $= \frac{11}{L}$.

The patient found it necessary to return home, and I directed his family physician to continue the treatment until four additional hypodermics had been given, at the end of which time patient called, with this marked improvement: R. E. $= \frac{21}{L}$, L. E. $= \frac{27}{L}$. The patient's business called him to the far West, and required his being away for four months. I gave him Hostelley's syrup of hydriodic acid to take during his absence. On his return the condition of hearing was almost normal, and remains the same at this writing.

As the details of a large number of such cases would consume much time and space, and withal prove tiresome, the object of this paper will have been accomplished by briefly stating that the writer has treated forty-seven cases of greater or less impairment of hearing—some amounting to almost entire deafness—by the method of hypodermic injections of pilocarpine muriate; and that from his observations in these cases he feels justified in expressing the following conclusions:

1. That age and sex have no influence on the success or failure of treatment.

2. That in recently developed deafness with tinnitus this treatment is much more hopeful of success than in cases of long duration. In one case, however, which was of specific origin, the patient, aged seventy-two years, had been almost deaf for twenty-two years, and yet this was one of the most successful cases treated. Nevertheless, this must be considered as very exceptional.

3. That cases of chronic suppurative otitis media, with some degree of impaired hearing, resulting from the exanthematous fevers, are not proper cases to receive benefit from this method of treatment.

4. That deafness, vertigo, and tinnitus, arising from syphilis, seem to be especially benefited by the subcutaneous injection of pilocarpine.

5. That these results can only be attained by pushing the pilocarpine to its full physiological effect, and that profuse diaphoresis must be obtained in every case.

---

### HOW TO USE MYDRIATICS.[1]

By EDWARD JACKSON, M. D.,

Professor of Diseases of the Eye in the Philadelphia Polyclinic; Surgeon to Willis Eye Hospital, etc.

The present purpose is to discuss methods, not indications, for using these drugs; but, in passing, it is worth repeating, since it is so often forgotten, that remedies of this sort are too powerful to be used indiscriminately. If one has not been able to make a positive diagnosis in a case of ocular inflammation, to clearly recognize the indications, and to definitely exclude the contra-indications for one of these drugs, he should let them alone, and confine his hit-or-miss prescribing to such agents as boric acid, or weak solutions of common salt, whose power for harm is really very slight.

These drugs are applied to the eye for their direct influence on the cornea, iris, or ciliary body. In either case they must be absorbed through the cornea, the lymph streams of which are in close relation with those of the anterior chamber. Any portion of the drug that may be absorbed from other parts of the conjunctival sac is carried into the general circulation without coming in

1. Read June 24, 1891.

contact with the structures it is intended to influence. Any solution placed in the conjunctival sac is almost immediately diluted by the lachrymal secretion present; only the part with which it first comes in contact receives it of full strength. Now, if the amount of fluid instilled is very large as compared with the amount of tears diluting it, the dilution is of very little importance. But instillations of large amounts of mydriatic solutions are not advisable, because they give the maximum of absorption into the general circulation with the minimum of effect on the eye. And one thing to be constantly guarded against in the use of mydriatics is the excess of constitutional action. Therefore, a mydriatic solution used in the eye should be instilled so as to come immediately in contact with the cornea while of full strength; that is, it should be placed at the upper margin of the cornea, allowed to flow over the surface of that membrane, and the closure of the lids prevented as long as possible, to allow absorption to occur before the fluid is swept away by the movements of the lids and diluted with the tears.

Instilled in this way, the concentration of the solution when it comes in contact with the corneal tissue, and consequently the amount absorbed, may be ten times as great as if the single drop of the same solution had been placed in some other part of the conjunctival sac. Thus applied, a very small drop of solution suffices to bathe the whole cornea. A dropper giving a small drop is, therefore, to be chosen. One is readily obtained with a small point that will drop half minims, or even less. The use of such a dropper allows the employment of stronger solutions than it would otherwise be safe to employ, or a larger number of instillations may be made in the same space of time without producing symptoms of mydriatic poisoning.

It is by attention to such a minute point of technique that one surgeon will at once secure the dilatation of an inflamed iris, or the complete relaxation of the accommodation under homatropine, where another less careful will fail to attain the end sought, or to give relief to his patient. And even where the utmost power of the mydriatic does not need to be exerted to obtain the effect that is required, with the least danger of constitutional symptoms, or with the minimum of constitutional disturbance, is a very important point; for these symptoms, although really not indicating any danger to life, are extremely annoying and alarming to the patient. They occur quite frequently after the use of mydriatic solutions, and such occurrence has much to do with the objection of patients to the use of mydriatics in the diagnosis of ametropia.

The strength of the solution of one of these drugs to be used in the eye varies with the purpose for which it is used. To break up the adhesions in a case of iritis, the stronger mydriatics are to be employed and in strong solution: As atropine sulphate, one to water fifty, or about ten grains to the fluid-ounce; daturine sulphate, one to water 100, or about five grains to the fluid-ounce; duboisine sulphate, one to water 100, or about five grains to the fluid-ounce; hyoscyamine sulphate or hydrobromate, one to water 100, or about five grains to the fluid-ounce. The effect of either of these solutions may be somewhat increased by using cocaine with it. But the patient should not be intrusted with the cocaine solution for home use, because the temporary comfort it gives, in many cases, leads sometimes to dangerous excess. Either of the above solutions is to be used one small drop in the eye at a time, at intervals of ten minutes, until the dilatation of the pupil is secured, and then at such intervals as may be necessary to maintain such dilatation; and continued three times daily until it can be replaced by a weaker solution.

In making the mydriatic attack on a case of plastic iritis, it is, to a certain extent, simply a question of whether we can get enough of the mydriatic into the eye without getting too much into the general circulation. And to accomplish this we must prevent the solution from making its way into the tear passages, and so being absorbed from the respiratory and digestive tracts, as well as from the conjunctiva. For this purpose it is often recommended to make pressure on the inner canthus. But such pressure is quite ineffective. Even the placing of a little clamp on each canaliculus, as proposed by Dr. Tansley, (*Trans. Amer. Ophthalmological Society*, 1888,) does good mainly by the displacement of the puncta that it causes. The most effective means is to so draw on the skin of the lids as to evert the puncta, and hold in contact with them a small pledget of dry absorbent cotton. This will prevent the passage of any fluid from the eye into the lachrymal sac, and permit us to apply the mydriatic vigorously to the cornea.

For paralyzing the accommodation of the eye, solutions of the same drugs of about half the above-mentioned strengths may be instilled three or four times daily.

Probably a single efficient instillation of this kind, or at most two or three, would be sufficient to produce complete paralysis of the accommodation in almost every case, with the eye in anything like normal condition. But frequently the instillation must be intrusted to unskilled hands, and so may produce but a small fraction of its full effect, and in a few cases the active hyperemia, caused by the mydriatic and involving the anterior segment of the globe, may increase the difficulty of attaining complete ciliary paralysis; so that it may be necessary to continue such applications for some days.

For simply paralyzing the accommodation, however, our most valuable agent is homatropine, commonly used in the form of the hydrobromate. Of this a two or three per cent. solution, ten or fifteen grains to the fluid-ounce, should be instilled every five or ten minutes until at least four efficient applications have been made. Used in this way, I have found it a perfectly reliable and efficient paralyzant of the accommodation, even in the presence of high grades of retino-choroidal irritation and general hyperemia of the eye. But we have not with this drug the excess, or reserve, of power to control the ciliary muscle, that is possessed by the other mydriatics named. Every instillation, or at least a sufficient number of them, must be efficient. The cornea must have the chance of absorbing the solution at nearly its full strength; and for that reason the application of the drug must be intrusted only to skilled hands, usually attended to by the surgeon himself.

To bring about simple dilatation of the pupil, our choice of the drug will be determined by whether the dilatation is to be long sustained as a measure of treatment, or only temporary as for purposes of diagnosis. In the former case, atropine is to be used; in the latter, homatropine or cocaine. Atropine or homatropine should be employed in a solution one-tenth the strength of those used for paralyzing the accommodation, or even weaker than this. The atropine to be repeated as often as the pupil contracts again, say once every one, two, or three days; the others, of course, used only the once.

Cocaine, which is of especial value as a dilator of the pupil, is to be used in solutions of the ordinary strength ordinarily employed for producing local anesthesia of the eye, that is, two to four per cent. But the instillation must be made at least thirty minutes, often an hour, before the dilatation is desired, the anesthetic action often having quite passed away before the dilatation of the pupil becomes noticeable, and repeated instillations do not very greatly hasten this dilatation. As a paralyzant of accommodation, cocaine has very little power, and by itself is not at all valuable for the purpose. But it can sometimes be advantageously combined with homatropine. Here the frequent repetitions of the instillation, as in the case of iritis, give the advantage of local anesthesia, greatly lessened resistance on the part of some patients, and prevention of the excessive secretion of tears that follows each instillation of homatropine alone, and by dilution of the solution lessens the intra-ocular effects produced, as well as an apparent hastening of absorption. For this purpose the solution may be made with two or three per cent. each of cocaine and homatropine.

The instillation of a strong solution of any of the mydriatics causes a pericorneal hyperemia, which, though not serious, is sometimes alarming to the patient or his friends. This phenomena I pointed out in a paper on homatropine, published in *The Medical News*, July 18th. It is especially liable to occur from the use of homatropine, because this is more likely to be used in stronger solutions. The combination with cocaine lessens this tendency to a considerable extent.

### DISCUSSION.

Dr. T. B. Schneideman: I wish merely to call attention to the change which takes place in mydriatics when kept in solution, from the formation of a precipitate due to the growth of a fungus. I do not think that this interferes with the efficiency of the solution, although it may increase the pain. We should also remember that when we intrust the mydriatic to the hands of the patient or his friends, we often fail to get complete paralysis of the accommodation. In hospital work we often find the mydriasis disappointing on this account.

---

### PETROLATUM.

Exhibited by Dr. John Aulde.

The subject of petrolatura was introduced into the Pharmacopeia of 1880 to cover several important products, such as cosmoline and vaseline. A considerable lack of knowledge exists in the minds of physicians in regard to the various petrolatum products. My object tonight is mainly to call attention to the appearances of these products. I have used petrolatum products largely, and have accumulated a number of specimens, which I shall present tonight. Petrolatum is extensively used for many purposes. It is largely used by veterinarians. It is used by actresses who first apply perfectly colorless cosmoline to the face and follow it with any desired powder. After the performance, the whole is washed off with a little cologne. It has been found that the colored petrolatum products produce discoloration of the skin. Cosmoline is perfectly innocuous, and may be taken into the system without harm. Through the kindness of Mr. Drill, the superintendent of a large factory where these products are made from crude petroleum, I had an opportunity of observing the processes. I was told by the workmen that when they have a bad cold they fill the nostrils with cosmoline and the trouble is quickly relieved.

These products are obtained by fractional distillation. The first twenty per cent. is called naphtha, and embraces several substances, such as rhigolene, benzine, naphthol, etc. The next fifty per cent. that passes over is composed of illuminating oil. This leaves about thirty per cent., fifteen of which is called neutral product, and fifteen per cent. called petrolatum stock. From this last, cosmoline is manufactured. The neutral product is decolorized by filtration through bone black. It contains a certain proportion of paraffine wax. This neutral product corresponds closely to teraline, which has been extensively advertised as a remedy for consumption. The paraffine is removed by crystallization and freezing. Teraline may be of great benefit, because it contains this wax. Suppose you have a case of inflammation of the bowels with distention of the capillaries and absorption of poisonous bodies, the use of an oil containing wax would act as a local protective, as does bismuth.

Here I show you a colorless product called glycoline, alboline, and several similar names. This is an oil with the paraffine and coloring matter removed. It is made both as a liquid and as a semi-solid substance. Here is the liquid paraffine of the German Pharmacopeia, which closely corresponds with our alboline. Here I have a number of preparations of petroleum stock, varying in color from white to dark yellow. Here is crude petroleum in various forms.

Here I have a section of lung tissue prepared in the following way: It is thoroughly washed with water, then with alcohol and de-alcoholized. A mixture of oil of cloves and oil of cedar, mixed in such proportions that a glass rod introduced into the mixture shows no angular refraction, is introduced into the lung tissue, and the whole covered with paraffine wax and a little resin, and then dipped into ice water. It can then be readily cut with a knife.

DISCUSSION.

Dr. S. Solis-Cohen: I use petroleum products in two ways—one internal, and the other as a vehicle for applications to the mucous membranes. Crude petroleum is very valuable in the treatment of pulmonary complaints, especially certain stages of phthisis. It is useful combined with iodoform. Two or three grains of iodoform may be combined with an equal quantity of crude petroleum and administered in capsules. So-called alboline, or a modification of it called benzonole, is useful as a vehicle for menthol and other agents, to be applied to the respiratory mucous membrane.

Mr. Drill: There is little to be added to what has been said by Dr. Aulde. Few physicians seem to know anything about the practical preparation of these products. The idea seems to be that cosmoline is a by-product. It never was a by-product. It is really the most valuable part of the petroleum distillation. The first seventy per cent. of the distillate is worth probably five cents a gallon, while the remaining thirty per cent. is worth from twelve to twenty-five cents a gallon. The quantity of these products has greatly increased. Where pounds were used ten years ago, tons are now used, and it is sent all over the world.

Dr. Robinson: I wish to refer to a single experience with liquid cosmoline. A man came to me with acute gonorrhea, and was anxious that something be done to relieve him at once. I injected about two drachms of liquid vaseline into the urethra. The symptoms at once began to subside, and the man had a very short attack—the second and third stages being absent. I do not know that the injection had anything to do with it, but I thought it well to relate this experience.

Dr. William F. Waugh: A few years ago an effort was made in France to introduce petroleum products as vehicles for the hypodermic use of remedies. It was shown that the purified oils were innocuous, as much as a kilogramme having been injected beneath the skin of a horse at one time without causing irritation. In some respects the experiments were of value. It was shown that in this way twenty minims of purified creosote could be introduced at a single dose. If creosote is useful at all in phthisis as a germicidal remedy, the advantage of giving so large a quantity would render this method of some importance.

Dr. James Collins: I well remember the time when the first specimen of a curious oil, said to come from the earth, was brought to the laboratory of.........., who proceeded to investigate it. He obtained naphthol, paraffine, and some other products from it. He brought the subject to the attention of some friends, who asked him if he had exhausted the oil. "No," he said, "the resources of chemistry would be exhausted by this agent." This was a long time ago, and the clang and clamor of the war drove the subject from my mind. When I returned to practice I began the use of these petroleum products, and since then cosmoline and alboline have been in my office constantly. I use them in nasal and throat affections. The only use in which I have been disappointed has been in phthisis. I have tried teraline faithfully, but it has failed to fulfil the promises made for it. As an internal remedy petrolatum has not been satisfactory, and it is probable that the digestion of this hydrocarbon is not as perfect as that of some other hydrocarbons.

Dr. Aulde: I have nothing further to add except to say that I purpose preparing a paper referring to the therapeutic indications of the petrolatum products. I believe that too much has been expected of this agent. The use of these products internally should be only as an assistant to other constitutional remedies They are not absorbed, and can only do good by their local effect.

